# Controlled spatial and conformational display of immobilised bone morphogenetic protein-2 and osteopontin signalling motifs regulates osteoblast adhesion and differentiation *in vitro*

**DOI:** 10.1186/1741-7007-8-57

**Published:** 2010-05-10

**Authors:** Elizabeth A Mitchell, Benjamin T Chaffey, Andrew W McCaskie, Jeremy H Lakey, Mark A Birch

**Affiliations:** 1 Institute for Cellular Medicine, The Medical School, Newcastle University, Newcastle-upon-Tyne, NE2 4HH, UK; 2 Institute for Cell and Molecular Biosciences, The Medical School, Newcastle University, Newcastle-upon-Tyne, NE2 4HH, UK

## Abstract

**Background:**

The interfacial molecular mechanisms that regulate mammalian cell growth and differentiation have important implications for biotechnology (production of cells and cell products) and medicine (tissue engineering, prosthetic implants, cancer and developmental biology). We demonstrate here that engineered protein motifs can be robustly displayed to mammalian cells *in vitro *in a highly controlled manner using a soluble protein scaffold designed to self assemble on a gold surface.

**Results:**

A protein was engineered to contain a C-terminal cysteine that would allow chemisorption to gold, followed by 12 amino acids that form a water soluble coil that could switch to a hydrophobic helix in the presence of alkane thiols. Bioactive motifs from either bone morphogenetic protein-2 or osteopontin were added to this scaffold protein and when assembled on a gold surface assessed for their ability to influence cell function. Data demonstrate that osteoblast adhesion and short-term responsiveness to bone morphogenetic protein-2 is dependent on the surface density of a cell adhesive motif derived from osteopontin. Furthermore an immobilised cell interaction motif from bone morphogenetic protein supported bone formation *in vitro *over 28 days (in the complete absence of other osteogenic supplements). In addition, two-dimensional patterning of this ligand using a soft lithography approach resulted in the spatial control of osteogenesis.

**Conclusion:**

These data describe an approach that allows the influence of immobilised protein ligands on cell behaviour to be dissected at the molecular level. This approach presents a durable surface that allows both short (hours or days) and long term (weeks) effects on cell activity to be assessed. This widely applicable approach can provide mechanistic insight into the contribution of immobilised ligands in the control of cell activity.

## Background

It has long been recognised that cell regulatory molecules, such as growth factors and cytokines, exert powerful influences on the behaviour of eukaryotic cells at the interface of tissues. Indeed the immobilized activity of these signalling mediators in combination with the extracellular matrix (ECM) underpins many fundamental biological processes including embryo-, morpho- and tumorogenesis and wound healing [1]. Numerous studies have established the principle that tethered ligands regulate cell behavior quite distinctly from their freely diffusible counterpart. For example, extracellular matrix proteins like fibronectin promote adhesion [2] and migration [3] on a surface but when added exogenously to cells can have adhesion-independent effects, for example activating intracellular signalling cascades [4]. Furthermore, soluble growth factors, like fibroblast growth factor (FGF)-2, vascular endothelial growth factor (VEGF) and interleukin (IL)-1 exhibit elevated activity when presented within the context of matrix such as fibrin [5-7]. Whilst these and many other studies have provided fundamental insight into the underlying biology, the experimental approaches have meant that immobilised ligands are often presented in orientations and conformations that are not well controlled, at densities that are poorly characterised and under conditions in which the durability and stability of the ligand is undefined.

Our approach has been to design and fabricate protein scaffolds that can display protein ligands on self-assembled monolayers (SAM) in a controlled and reproducible way [8,9]. This work uses a TolAIII-fusion expression system to provide the engineered protein at high yields and solubility [10]. A cysteine residue engineered into the C-terminal end of the protein ensures durable chemisorption onto gold surfaces whilst a 12 amino acid stretch termed the *switch-tag *(ST) can switch from a water soluble coil to a hydrophobic helix that co-assembles with the alkane thiols of the SAM [8]. Therefore formation of a SAM containing both the scaffold protein and an amphiphile produces a surface where the orientation of the protein is controlled and non-specific interactions are low. These surfaces provide the opportunity to address how immobilised ligands can both provide insights into cell function and contribute to tissue engineering.

We demonstrate here the ability of this system to influence the long term function of cells *in vitro *via the incorporation of two distinct ligand motifs. The first is derived from the extracellular matrix protein, osteopontin (OPN), and was identified as supporting cell adhesion through the α_9_β_1_ integrin [11] and subsequently shown to promote adhesion and migration of endothelial cells leading to enhanced angiogenesis [12-14]. The second is the *knuckle epitope *from bone morphogenetic protein (BMP)-2, a member of the transforming growth factor (TGF)β superfamily, which has been widely shown to support commitment of cells to the osteoblastic lineage, their differentiation and functional ability to make bone [15,16].

Here we show that Tol-OPN-ST dose-dependently supported the adhesion and spreading through vinculin adhesion sites of primary rat osteoblasts. Biological activity of the BMP motif within the scaffold protein was verified by adding the recombinant protein to cells transfected with a Sma- and MAD-related protein (SMAD) responsive-reporter construct and demonstrating SMAD1 activation. Tol-BMP2-ST reproduced BMP signalling and produced bone-like nodules on patterned surfaces.

This technology effectively immobilises bioactive protein motifs on a surface for analysis of cell behaviour *in vitro *and may provide the basis for future tissue engineering approaches.

## Results

### Incorporating osteopontin or BMP2 motifs in the Tol scaffold protein does not influence its binding to gold

**Figure 1:**
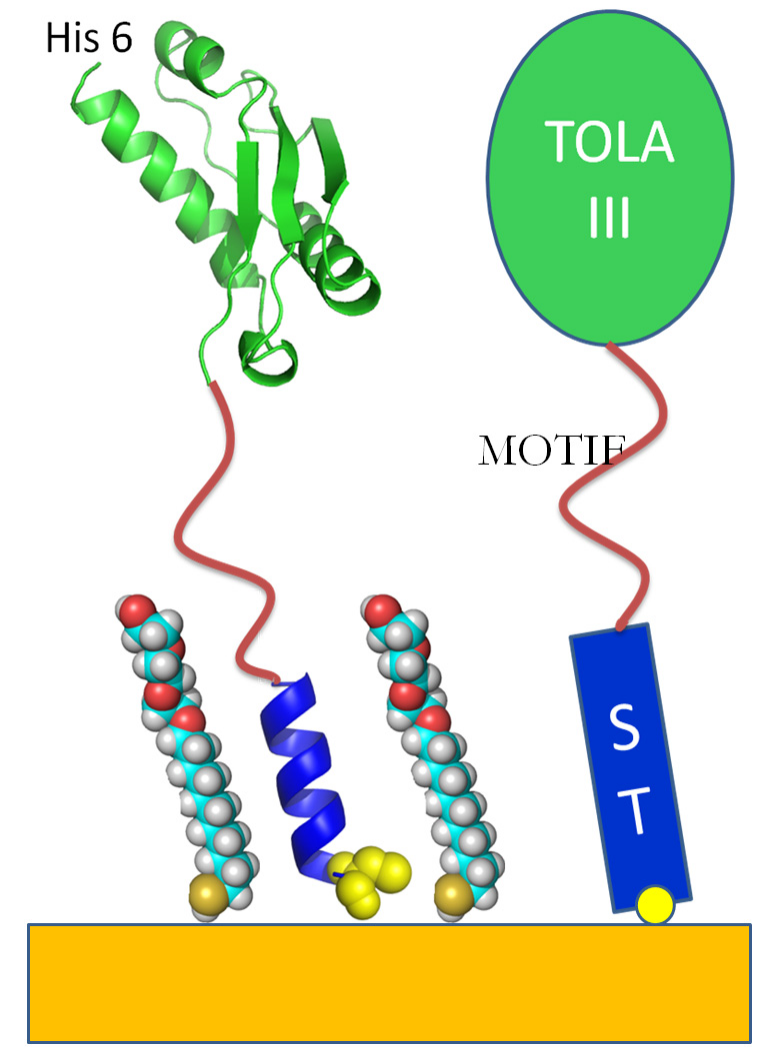
**The design of the Tol switch tag system, a molecular image (left) and a schematic (right)**. The protein starts at the N-terminus with a 6 His tag for easy purification followed by a FLAG-tag sequence to aid detection. The TolAIII domain which comes next provides solubility and drives high levels of expression for fusion proteins attached to its C terminus [10]. The fusion sequence is inserted between the TolA and the switch tag, which is found at the C terminus, by use of a multiple cloning site in the plasmid [8]. The TolA domain can be removed by specific proteolytic cleavage but in most cases it is left in place since it stabilises the fusion construct. The switch tag is shown here as a helix whose hydrophobic properties allow co assembly with alkane thiol SAM, in solution it is a water soluble random coil.

The motifs from osteopontin and BMP2 were inserted between Tol and the Switch tag to create Tol-OP-ST and Tol-BMP-ST respectively (Figure 1). This design ensured that the C-terminal cysteine was available to bind gold surfaces and that the biological activity of the inserted motifs is not compromised. BIAcore analysis (Table 1) shows that Tol-OP-ST and Tol-BMP-ST specifically interact with the gold surface and, interestingly, binding of Tol-OPN-ST and Tol-BMP-ST was slightly higher than Tol-ST alone. The vector includes a protease cleavage site which can remove the Tol protein leaving just OPN-ST or BMP-ST. However since the larger construct is more stable and easier to handle in solution, the initial studies used the complete fusion protein

**Table 1:** SPR data of the Tol, Tol-Osteopontin (Tol-OP) and Tol-BMP proteins binding to a gold surface in SPR analysis (BIAcore).

Protein	rU increase	Mr of protein	moles/mm^2^	Molecules/mm^2^
Tol	1,526.53	1,4874.4	1.03 × 10^-13^	6.4 × 10^10^

Tol-Op	4,381.15	18,407.3	2.38 × 10^-13^	14.8 × 10^10^

Tol-BMP	3,162.26	17,894.8	1.77 × 10^-13^	11.0 × 10^10^

The proteins were added at a concentration of 5 mM in a total volume of 30 μl over a period of 15 minutes.

### Cells specifically interact with immobilised Tol-OPN-ST

**Figure 2:**
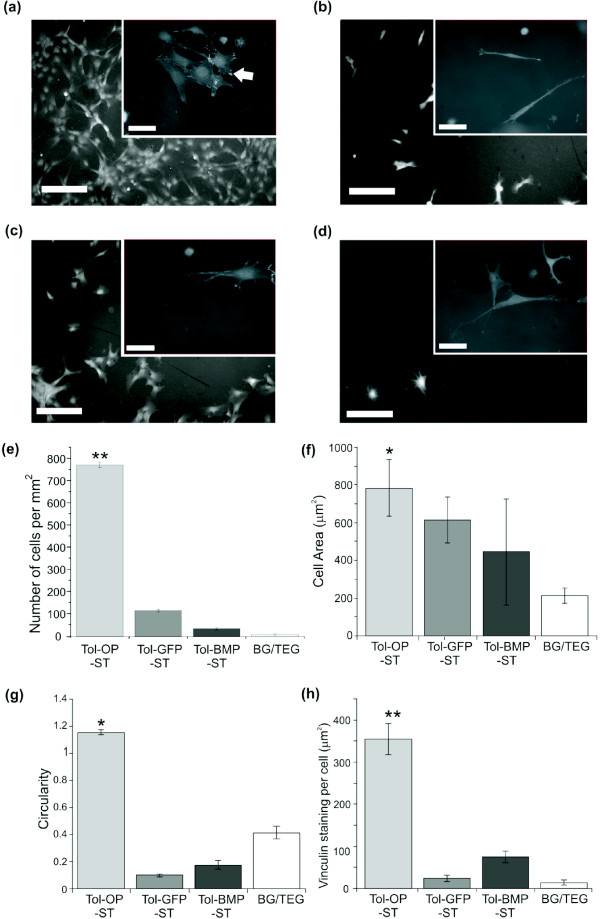
**Immobilised Tol scaffold containing the osteopontin motif, SVVYGLR, specifically controls cell adhesion and cell morphology**. Cells were plated onto surfaces displaying scaffold proteins in the absence of serum supplements and assessed after 24 hours. Immunolocalization of vinculin and counterstaining with DAPI allowed identification of focal adhesion complexes (white arrow) in cells on **(a) **Tol-OPN-ST; **(b) **Tol-GFP-ST; **(c) **Tol-BMP-ST; **(d) **TEG. Size bars = 200 μm and 50 μm in the inset images. Image analysis demonstrated significant differences in **(e) **the number of cells adhered to Tol-OPN-ST compared to all other surfaces; **(f) **the area of cells on Tol-OPN-ST compared to TEG; **(g) **the circularity of cells on Tol-OPN-ST compared to the other surfaces and **(h) **abundance of vinculin positivity in cells on Tol-OPN-ST compared to other surfaces. Data representative of three independent experiments; results are mean ± SEM; *P < 0.05, ** *P *< 0.01. Significance was determined by ANOVA with Fisher post test.

Having demonstrated that the proteins chemisorb to the gold surface irrespective of the active motif inserted in the scaffold, the ability of the cells to interact with these immobilised proteins under serum-free conditions was determined (Figure 2a, b, c, d). After 24 hours of culture Tol-OPN-ST promoted the attachment of significant (*P *< 0.01) numbers of cells compared to all other surfaces (Figure 2e). Analysis of cell morphology revealed that on Tol-OPN-ST, area (Figure 2f) was significantly (*P *< 0.05) enhanced compared to bare gold/11-mercapto-1-triethyleneglycolundecane (TEG-thiol) whilst circularity (Figure 2g) was significantly (*P *< 0.01) different from all of the other surfaces that were studied. In addition, around the periphery of the cells there were focal contacts, characterised by the identification of the protein vinculin by immunofluorescence. These contacts are important since this is where the cell interacts with the extracellular matrix (Figure 2a) with significantly (*P *< 0.01) increased abundance of vinculin in cells on Tol-OPN-ST compared to all of the other surfaces (Figure 2h). Cells on the Tol-OPN-ST surface also exhibited a more spread out morphology that correlates with a more robust cytoskeleton consisting of numerous actin filaments (Figure 2f, g). Relatively few cells were observed to adhere to the surfaces displaying immobilised Tol-BMP2-ST (Figure 2c, e) or Tol-green fluorescent protein (GFP) (Figure 2b, e), indeed these surfaces were little different to surfaces composed solely of the anti-adherent TEG-thiol (Figure 2d, e). Furthermore those cells that grew on Tol-BMP, Tol-GFP and control TEG alone formed fewer focal contact structures and were less well spread on the surfaces (Figure 2f, g, h).

**Figure 3:**
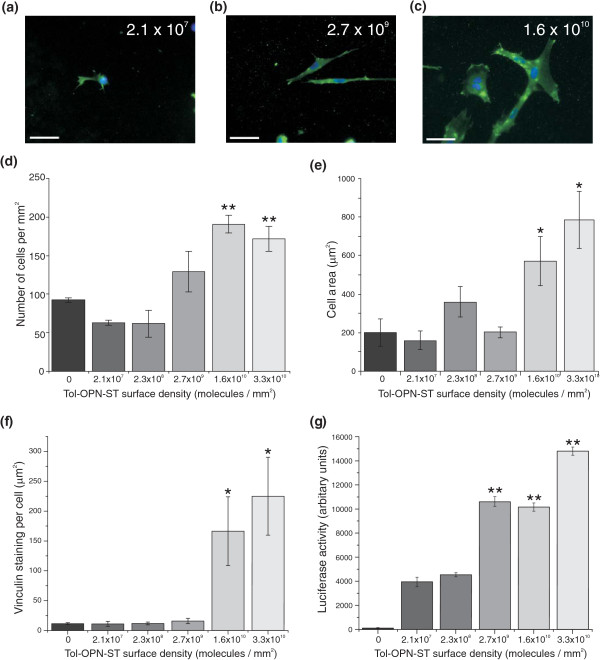
**Tol-OP-ST surface density influences cell morphology and response to BMP2**. Gold coverslips were incubated with different concentrations of Tol-OP-ST and based on SPR analysis surface densities calculated (molecules/mm^2^). **(a, b, c) **Cells were plated onto the surfaces, as indicated, under serum free conditions and vinculin immunolocalized 24 hours later. Size bars = 50 μm. **(d) **DAPI was used to visualize nuclear DNA and images quantified to show number of adhered cells. Numbers of cells on 1.6 and 3.3 × 10^10 ^molecules/mm^2 ^were significantly different to all other surfaces. **(e) **Assessment of cell area revealed that cells on 1.6 and 3.3 × 10^10 ^molecules/mm^2 ^were significantly different to all other surfaces. Furthermore **(f) **levels of vinculin positivity were significantly higher in cells on 1.6 and 3.3 × 10^10 ^molecules/mm^2 ^in comparison to lower densities. **(g) **Cells transfected with a SMAD-reporter construct were plated on the surfaces, treated with 100 ng/ml recombinant BMP-2 and levels of luciferase assessed and normalised to cell number. Data representative of three independent experiments; results are mean ± SEM; * *P *< 0.05, ** *P *< 0.01. Significance was determined by ANOVA with Fisher post test.

Next we investigated cell adhesion on surfaces with varying densities of immobilised scaffold (Figure 3a, b, c). Scaffold was immobilised at different densities by adjusting the coating concentration of protein. The number of cells binding to the osteopontin surface was dependent on the surface density of Tol-OP-ST (Figure 3d) and this correlated closely with both cell area (Figure 3e) and the abundance of vinculin-containing focal contact structures (Figure 3f). Analysis of cell morphology on these surfaces demonstrates the influence of Tol-OPN-ST density on cell behaviour. At relatively low density (2.1 × 10^7 ^molecules/mm^2^) Tol-OPN-ST surfaces promote poor cell adhesion and limited cell spreading. While at a high density (1.6 and 3.3 × 10^10 ^molecules/mm^2^), Tol-OPN-ST supports significant cell adhesion (*P *< 0.01), extensive cell spreading (*P *< 0.05) and the formation of significantly more abundant and complex cytoskeletal elements (*P *< 0.01). At an intermediate surface density (2.7 × 10^9 ^molecules/mm^2^) (Figure 3b), cell adhesion is more weakly promoted with cellular processes extended but not sufficiently strengthened to facilitate cell spreading. To further illustrate the biological specificity of our approach we investigated the cellular response to soluble recombinant BMP2 (srBMP2) when cultured on surfaces displaying different densities of Tol-OPN-ST. It is widely appreciated that integrin-mediated adhesion influences the regulation of growth factor induced signalling cascades and indeed this phenomenon is believed to underpin numerous biological events such as morphogenesis and wound healing [17]. For this study, osteoblasts were transfected with a luciferase reporter construct driven by SMAD-binding elements and cultured on different densities of immobilised Tol-OP-ST for eight hours under serum-free conditions. The cells were then treated with soluble recombinant BMP2 and the response assessed through analysis of luciferase activity (Figure 3e). Cells that were more extensively spread on higher densities of Tol-OP-ST exhibited significantly (*P *< 0.01) higher levels of BMP-induced signalling compared to cells that were poorly spread and showing fewer adhesion complexes on low density Tol-OP-ST. This confirms that BMP receptor activation is influenced by the density of ECM ligands.

### Osteoblasts respond specifically to both soluble and immobilised Tol-BMP-ST

**Figure 4:**
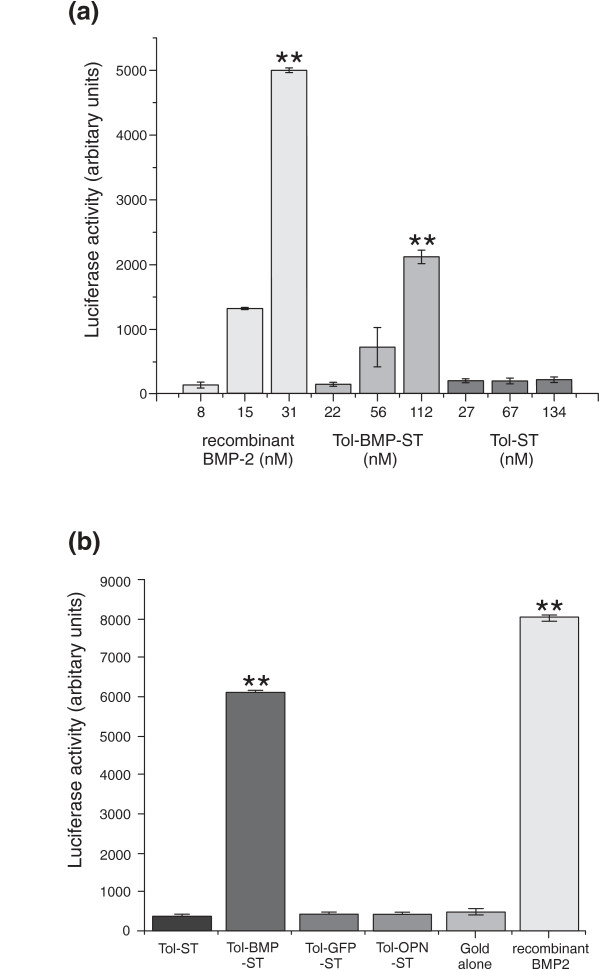
**Tol scaffold containing the BMP2 *knuckle *peptide, KIPKASSVPTELSAISTLYL, activates Smad dependent signalling both when added exogenously to adherent cells and when immobilised on a surface**. **(a) **Cells were cultured in multiwell plates, transfected with a SMAD-reporter construct and then treated with recombinant BMP2, exogenous Tol-BMP-ST or Tol-ST. Levels of luciferase were then assessed after eight hours. Both recombinant BMP2 (31 nM) and Tol-BMP-ST (112 nM) induced significant levels of luciferase activity compared to Tol alone. **(b) **Cells already transfected with the SMAD-reporter construct were plated onto gold coverslips displaying the scaffold proteins at the concentrations outlined in Table 1 or TEG alone. Luciferase activity was measured 12 hours later and contrasted with the same cells grown on tissue culture plastic and treated with soluble recombinant BMP-2 (100 ng/ml). Significant levels of SMAD signalling were only observed on the Tol-BMP-ST surface. Data representative of three independent experiments; results are mean ± SEM; ** *P *< 0.01. Significance was determined by ANOVA with Tukey post test.

To test the ability of Tol-BMP-ST to activate BMP-dependent intracellular signalling, it was added exogenously to cells growing on conventional tissue culture plasticware. Analysis reveals that cells transfected with SMAD-binding promoter construct were dose-dependently responsive to both soluble recombinant BMP2 and Tol-BMP-ST (Figure 4a) with the highest doses significantly different from Tol-ST alone (*P *< 0.01). The cells exhibited no response above background to the Tol protein vector alone. Next, cells transfected with the same reporter construct were plated onto specific Tol-X-ST surfaces (Figure 4b). Only those cells grown on the Tol-BMP-ST surface demonstrated activation of the SMAD signalling pathway that was comparable to the effect of soluble recombinant BMP. Levels of SMAD signalling observed on both Tol-BMP-ST surfaces and in those cells treated with recombinant BMP2 were significantly different (*P *< 0.01) compared to all other surfaces.

### Immobilised Tol-BMP-ST induces enhanced osteogenesis *in vitro*

**Figure 5:**
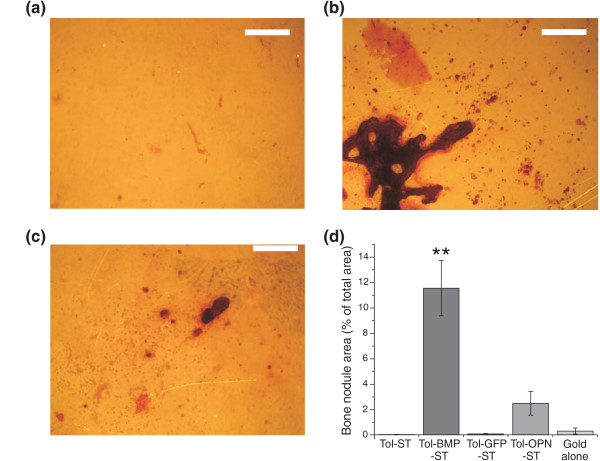
**Long term growth of calvarial-derived cells on scaffold proteins**. After four weeks under routine culture conditions and in the absence of exogeneous osteogenic factors mineralized matrix was visualized with Alizarin red staining. There was little evidence of mineralised matrix on **(a) **Tol alone, whilst abundant deposition was observed on **(b) **Tol-BMP-ST and a small amount on **(c) **Tol-OP-ST surfaces. Size bar = 400 μm. **(d) **Image analysis shows significant evidence of mineralised matrix deposition only on Tol-BMP surfaces. Data representative of three independent experiments; results are mean ± SEM; ** *P *< 0.01. Significance was determined by ANOVA with Tukey post test.

Tol-BMP-ST, Tol-OPN-ST and Tol-ST were then compared for their ability to influence the long term behaviour of cells *in vitro*. Primary rat osteoblasts can be cultured in media supplemented with β-glycerophosphate, L-ascorbic acid and dexamethasone to promote osteogenic differentiation over a period of weeks [18,19]. In the absence of this chemical cocktail the cells retain a more primitive fibroblastic phenotype. In our experiments, cells were maintained in culture without these soluble supplements so that the only potential osteogenic signal was from the engineered surfaces. In this environment cells proliferated and achieved confluence on all surfaces after three days. After two weeks of culture there was clear evidence of cells on Tol-BMP-ST surfaces beginning to form multilayers and discrete cellular aggregates. This behaviour was not repeated on Tol-OPN-ST, Tol-ST or other control surfaces. After four weeks, in the absence of further osteogenic factors, extensive bone-like nodule formation was observed on Tol-BMP-ST (Figure 5b, d) which was significantly (*P *< 0.01) more extensive than on the other surfaces investigated. There was some evidence of mineralised matrix production on Tol-OP-ST (Figure 5c) whilst on Tol-ST alone (Figure 5a) and bare gold there very few bone-like nodules (Figure 5d).

### Surface patterns of Tol-BMP-ST induce spatially restricted osteoblast differentiation

**Figure 6:**
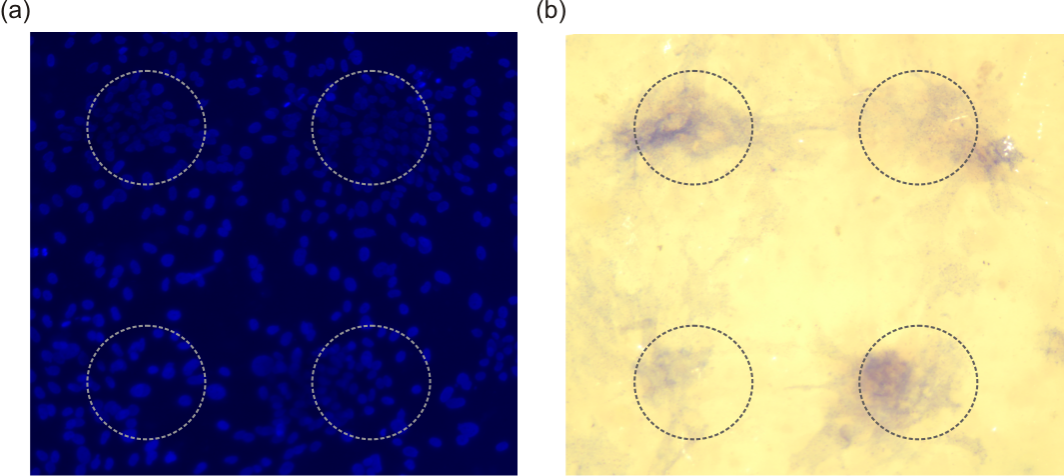
**Micro-patterned Tol-BMP-ST supported spatially restricted differentiation of calvarial-derived cells**. Tol-BMP-St was patterned onto gold surfaces using a soft lithography approach to create 150 μm diameter circles of scaffold protein. Cells were cultured on these surfaces for 14 days. **(a) **Cell distribution was assessed by DAPI staining for nuclear DNA and **(b) **cell differentiation was investigated by alkaline phosphatase staining (blue).

Whilst osteoblasts displayed enhanced differentiation when cultured on surfaces displaying Tol-BMP-ST it was not clear if Tol-BMP-ST was acting after desorption from the surface. To address this, patterned surfaces were created using a microcontact printing approach [8]. A polydimethylsiloxane (PDMS) stamp displaying microscale features was *inked *with Tol-BMP-ST and used to transfer a protein pattern to the gold surface. The gaps between the protein and between patterns were backfilled by incubation of the whole surface with TEG-thiol. Osteoblasts were cultured on these surfaces for two weeks and differentiation assessed by staining for alkaline phosphatase activity (Figure 6b). During two weeks cells grew all over the surface both in regions that were stamped with the Tol-BMP-ST pattern and those that were not (Figure 6a). The marker of osteoblast differentiation, alkaline phosphatase was principally localised to the regions where Tol-BMP was immobilised (Figure 6b). These experiments demonstrated that bioactive signalling was restricted to the patterned areas suggesting that surface tethered ligand was predominantly responsible for the regulation of cell activity and not Tol-BMP-ST released from the surface.

## Discussion

Signalling by interfacial motifs is a fundamental biological mechanism that underpins numerous aspects of morphogenesis and wound healing [1]. Tools that enable further insight into how immobilized ligands regulate cell behaviour will not only provide mechanistic understanding but also the basis for therapeutic approaches such as tissue engineering [20]. Our results show that a generic immobilisation system with a variable motif sandwiched between expression enhancing and self assembly domains (Tol-motif-ST) can present the motifs in a functional state at the surface of a SAM. The fusion protein is easily produced in large amounts in *E coli *and is easy to convert from a highly water soluble state to a SAM inserted form on gold [8]. Analysis by SPR of the Tol-ST proteins on a gold surface interestingly provided evidence that Tol-OPN-ST and Tol-BMP-ST pack at higher surface densities than Tol-ST alone. This may arise from flexibility introduced into the protein by the inclusion of the short OP and BMP motifs. The highest flexibility is expected from the OP domain as there are three linker sequences found in this sequence whilst the BMP motif is shorter. In addition analysis of Tol-GFP (data not shown) assembly by SPR shows this rigid construct to be similar to Tol alone, supporting the possibility that protein flexibility plays a role in its packing on the surface. Thus retention of the N terminal Tol domain does not inhibit self assembly and likely serves to solubilise and protect the flexible peptides from degradation, presenting the biologically active motifs in a more constrained structure.

The biological activity of the Tol-ST surfaces was first determined in simple cell adhesion assays performed in the absence of serum. The abundant cell adhesion on Tol-OPN-ST, in contrast to the other surfaces, is most likely mediated by α_9_β_1 _integrin as described for endothelial cells [11-14]. Calvarial derived osteoblasts have not been previously shown to express α_9_β_1 _integrins but they can be found on periodontal ligament cells [21] and cells of the osteoclast lineage [22]. The adhesion and spreading of cells described here is a possible demonstration of α_9_β_1 _integrins activity in these cells but an alternative mechanism cannot be discounted.

Surface assembly techniques provide the ability to control surface density of cell interaction motifs. Reducing the apparent density of Tol-OP-ST had significant effects on cell shape, morphology and numbers of vinculin-positive focal contacts. This revealed a threshold level of Tol-OP-ST below which cytoskeletal tensioning and remodelling cannot be supported (2.7 × 10^9 ^molecules/mm^2^), and above which the cells can interact with the surface initiating morphological change and regulation of cell behaviour. The estimated density measured here compares with other approaches that have attempted to address this issue where cells of fibroblastic origin exhibited increased cell adhesion and spreading on integrin ligands of the order 10^9 ^molecules/mm^2 ^[23-25]

The way in which cells adhere and interact with the surrounding matrix is known to not only influence cell shape but also to control cell response and gene expression [17]. Indeed this has been shown to be a mechanism that is important in the regulation of osteoblast differentiation [26,27] and response to BMPs [28-30]. To demonstrate that Tol-ST surfaces could be used to probe this phenomenon, different densities of Tol-OPN-ST were characterised for their ability to support BMP dependent signalling. A SMAD-responsive reporter construct showed significantly greater activation in response to srBMP-2 when cells were cultured on Tol-OPN-ST at 2.7 × 10^9^-3.3 × 10^10 ^molecule/mm^2 ^compared to 2.3 × 10^8 ^molecules/mm^2 ^and lower. It is particularly noteworthy that the apparent threshold for formation of abundant cell adhesion plaques (1.6 × 10^10 ^molecules/mm^2^) is higher than that for BMP receptor activation and signal transduction (2.7 × 10^10 ^molecules/mm^2^). This suggests that the mechanisms regulating BMP receptor signalling may well be independent of cytoskeletal organisation controlled by integrin adhesion to Tol-OPN-ST. On other substrates, α_v_β integrins [29] and Focal Adhesion Kinase (FAK) [31] have both been implicated in the osteoblastic response to BMP. Our results support general matrix-dependent BMP signalling in terms of FAK activity since weakly adherent cells demonstrated poor BMP responses whilst well adhered and spread cells exhibited elevated BMP effects. Nevertheless this does not exclude a role for integrin subunit specific responses with BMPRI and BMPRII directly associating with α_v _and β_1 _integrins [29], on appropriate external ECM ligands, to give altered responses.

Tol-BMP-ST maintained BMP-like activity and like srBMP-2 was able to activate the Smad signalling cascade. This is in agreement with the work of Saito *et al *(2003) who showed that a synthetic BMP2 *knuckle *peptide was able to induce alkaline phosphatase activity in C2C12 cells but less effectively than srBMP2 [16]. Previous work [32,33] has shown that immobilised BMP2 more effectively activates differentiation of osteoblasts than soluble BMP2. Tol-BMP-ST was immobilised on gold surfaces, back-filled with TEG-thiol and then compared with soluble recombinant BMP-2 for the ability to regulate osteoblast activity and bone formation. Immobilized Tol-BMP-ST showed activation of SMAD responsive elements driving luciferase expression in transfected cells. Interestingly the level of activation of signalling by the immobilised ligand was comparable to soluble recombinant BMP2, demonstrating its enhanced activity compared to soluble Tol-BMP-ST. The activity of this BMP2 *knuckle *peptide is postulated to be mediated through its interaction with BMP receptors I and II (BMPRI and BMPRII) [16]. Activation of preformed or induced BMPR complexes by soluble BMP results in initiation of intracellular signalling cascades that include the Smad pathway. In addition, BMPR complexes are believed to be internalised by the cell in processes which both alter the signalling dynamic and lead to loss of or attenuation of activation [34-38]. It is therefore likely that Tol-BMP-ST surfaces facilitate prolonged activation of BMP signalling since the receptor complex would be retained at the cell surface by the immobilised ligand and prevented from trafficking into the cell for example via lipid rafts. This is supported by the work of others which shows that immobilised recombinant BMP2 causes prolonged activation of osteogenic gene expression and enhanced osteoblast differentiation [39,40].

When cells were plated on Tol-BMP-ST under routine culture conditions, cell differentiation occurred during long term experiments with evidence of the formation of abundant bone-like nodules. In contrast, few mineralised matrix deposits were observed on Tol-OPN-ST and bare gold. Immobilised BMP has previously been shown to support osteogenic differentiation *in vitro *[32,40,41] using MC3T3-E1 cells and BMP2 that was immobilized by a heat treatment process. Data presented here use primary cells that are a heterogeneous population that retain the potential to differentiate to several different lineages but this is the first time that a tethered ligand alone and in the complete absence of other agents like dexamethasone not only induces ostegenic differentiation but also extensive deposition of a mineralized bone-like matrix. This illustrates the enhanced and sustained signalling provided by the Tol-BMP-ST surface reinforced cell regulator cues that drive the temporal control of cell phenotype. Furthermore Tol-BMP-ST on gold reproduces the asymmetric directional control of bone formation observed *in vivo*, a process that is believed to be guided by immobilised ligands. The formation of bone-like nodules *in vitro *requires extensive cell motility with the formation of three-dimensional cell aggregates and therefore the immobilised signal derived from Tol-BMP-ST is likely to be relayed to cells away from the surface by cell:cell interactions and paracrine signalling.

Spatial control of osteoblast differentiation was demonstrated by using microcontact printing of Tol-BMP-ST. This approach has been used widely in cell culture studies [42] with predominantly ECM motifs but this is the first time that cell differentiation motifs have been presented in this way. This showed that it is immobilised Tol-BMP-SP and not ligand lost from the surface that is directing cell behaviour. Work using an inkjet approach has recently reported similar findings with recombinant BMP2 spotted on a fibrin layer [43] illustrating the potential for patterns of immobilised biological ligands to control cell behaviour in a way that is spatially restricted.

## Conclusions

Understanding the molecular mechanisms of cell interactions and their regulation by surrounding matrix cues has important implications for biotechnology (production of cells and cell products) and medicine (tissue engineering, prosthetic implants, cancer and developmental biology). For example, it provides the basis for fabrication of scaffolds bearing biomimetics and/or therapeutics to facilitate tight control of cell phenotype in a target tissue. The technology described here, which immobilises recombinant proteins, is a highly controlled, adaptable and reproducible approach that allows the creation and screening of surfaces displaying bioactive cell interaction motifs. We have shown that this approach can provide an insight into the role of immobilised ligands in regulating cell behaviour, the interplay with soluble factors and the influence of spatially controlled signalling. Furthermore, this approach presents a durable surface that allows both short (hours or days) and long term (weeks) effects on cell activity to be assessed. Therefore this technology will considerably enhance current approaches that utilise cell-scaffold technology and also undoubtedly provide significant new data and therapeutic applications.

## Methods

### Production of recombinant proteins in *E. Coli *and their immobilization on gold surfaces

TolAIII-Switch tag fusion protein expression constructs (Tol-*motif*-ST) were based on the pTol-T vector, expressed in *E. coli *BL-21 cells and purified as detailed previously (Anderluh *et al *2003). The motifs derived from osteopontin (SVVYGLRGSGSGS) and BMP-2 (KIPKASSVPTELSAISTLYL) were engineered into the expression construct using a synthetic oligonuleotide approach. The potential for recombinant proteins to interact with a gold surface was evaluated using Surface Plasmon Resonance (SPR). The gold surface was cleaned with Piranha solution for 30 minutes, rinsed with H_2_O, 1% (w/v) sodium dodecyl sulphate (SDS) and finally extensively with H_2_O before drying with air and immediate docking into the Biacore X system. A buffer of 200 mM NaCl, 0.5 mM EDTA, and 20 mM Tris pH 8.0 was used for all solutions and also as the running buffer. The solutions used for thiol binding steps were supplemented with 0.1 mM TCEP (Tris(2-carboxyethyl)phosphine hydrochloride) for one hour before use. SPR analysis was used to calculate protein surface density (molecules/mm^2^) based on the observation that an increase of 1 rU equates to 1 pg/mm^2^. Molecules/mm^2 ^could then be found from rU increase × A/(Mr protein × 10^12^), where A = Avogadro's number. In all experiments, except where indicated, the Tol-*motif*-ST proteins were used to prepare surfaces with the densities outlined in Table 1. Surfaces for cell culture were prepared as above on gold-coated coverslips but were finally then immersed in 0.5 mM TEG-thiol (11-mercapto-1-triethyleneglycolundecane, HSC11-EG3) (ProChimia Surfaces, Sopot, Poland) for four hours to complete the formation of a surface monolayer.

### Microcontact printing on cleaned gold coverslips

CAD software was used to produce a five-inch mask design outlining individual 64 mm^2 ^patterned dies. The mask was manufactured by Delta Mask Bv (Enschede, Netherlands) to a tolerance of 0.35 μm. A patterned silicon wafer was fabricated from the mask using standard photolithographic techniques to create a photoresist master. The mold was silanized by leaving overnight in chlorotrimethylsilane vapour. Polydimethylsiloxane (Dow Corning, MIDLAND, MI, USA) was prepared as directed by the manufacturer and poured to a depth of 5 mm over the photoresist master, the chamber was degassed and left overnight to cure. Final cure of the PDMS was achieved by placing in an oven at 60°C for one hour. Stamps were cut from the PDMS layer with a scalpel and gently peeled away from the photoresist. In the experiments described here a stamp was used that created circles 150 μm in diameter within a 300 μm square array. The polydimethylsiloxane (PDMS) stamps were rinsed in water to remove any debris, and then immersed in ethanol and dried in a stream of compressed air, before being *inked *using recombinant Tol-BMP-ST (5 mM) and then dried in a stream of compressed air. The stamps were then placed on the surface of the Piranha-treated gold, left for 15 seconds and then the gold surface allowed to dry for 10 minutes. The gold surfaces were then immersed in 0.5 mM TEG-thiol (11-mercapto-1-triethyleneglycolundecane, HSC11-EG3) (ProChimia Surfaces, Sopot, Poland) for four hours.

### Cell isolation and culture

Culture media and supplements were obtained from Invitrogen (Paisley, UK) unless otherwise stated. Primary rat osteoblasts were isolated from three-day-old rat (Sprague Dawley) calvariae (Charles River UK Ltd, Margate, Kent, UK) based on methods previously described [18]. Cells were grown in Dulbecco's modified Eagle's medium (DMEM) supplemented with 10% fetal calf serum (FCS), 100 μg/ml streptomycin and 100 U/ml penicillin (all Invitrogen Ltd, Paisley, UK) at 37°C in a humidified atmosphere with 5% CO_2_. Cells were used up to passage 2 and subcultured using routine tissue culture approaches.

### Immunofluorescence

Cells at 70 to 80% confluence were trypsinised and resuspended in complete media. Cells were then seeded into six well plates containing gold-coated coverslips displaying the appropriate SAMs and incubated for 24 hours at 37°C in a humidified atmosphere with 5% CO_2_. Cells were then rinsed with PBS (Becton Dickinson UK Ltd, Oxford, UK) and fixed with 4% (w/v) paraformaldehyde in PBS for 15 to 20 minutes. Cells were permeabilised by incubation for five minutes in 0.1% Triton X-100 (v/v) in PBS and incubated for one hour at room temperature in 1:200 dilution of monoclonal mouse anti vinculin antibody (V9131 - Sigma-Aldrich Company Ltd, Gillingham, Kent, UK)). Samples were rinsed with PBS and incubated with a FITC-goat anti-mouse antibody (Zymed Laboratories Invitrogen Ltd, Paisley, UK). Following a wash step, cells were overlayered with VectaShield containing DAPI (Vector Laboratories Ltd, Peterborough, UK) and mounted for microscopy. The samples were then visualized on a Leica fluorescence microscope and images captured using Spot cooled CCD camera and software with digital images being analysed using ImageJ (Freeware). In analysis of cell morphology, circularity was defined as 4π(area/perimeter^2^).

### BMP responsiveness of cells

A luciferase reporter construct that was under the transcriptional control of 6× SMAD binding elements was used to demonstrate BMP induced intracellular signalling cascade [44]. Cells were seeded overnight to 50 to 60% confluency and then transfected with the reporter construct using Fugene (Roche, Burgess Hill, UK). Cells were then either plated onto the SAMs and luciferase activity assessed at the time points indicated or transfected cells were treated with soluble recombinant protein and response investigated. Luciferase activity was measured using the Bright-Glo™ Luciferase Assay System (Promega UK Ltd, Southampton, UK) and an automated microluminometer (MicroLumat Plus, Berthold Technologies (UK) Ltd, Harpenden, UK).

### Mineralised matrix and alkaline phosphatase staining

Alizarin red detects the presence of calcific deposition within the matrix by binding calcium ions to form a complex that is intensely red. Cells were washed in PBS and incubated in 1% (w/v) Alizarin red (pH 4.0) solution for 20 minutes in the dark before a final wash in distilled water. Cells on surfaces were then mounted onto glass slides and mounted in Vectashield with DAPI (Vector Laboratories Ltd, Peterborough, UK). To visualize alkaline phosphatase activity cell monolayers were fixed for 10 minutes in 4% (w/v) paraformaldehyde in PBS and then incubated in napthol AS-X phosphate and Fast Blue RR (pH8.4).

### Statistical Analysis

In all experiments data was analysed by ANOVA using either a Fisher or Tukey post test. Statistical significance was considered at *P < 0.05 and **P < 0.01. Experiments are presented as representative or at least three independent experiments: for analysis of cell number n = 8; for investigation of area, circularity and vinculin staining n = 20; for luciferase assays n = 6.

## Abbreviations

BMP: bone morphogenetic protein; DAPI: 4',6-diamidino-2-phenylindole; DMEM: Dulbecco's modified Eagle's medium; FGF: fibroblast growth factor; IL: interleukin; OPN: osteopontin; PBS: phosphate buffered saline; PDMS: polydimethylsiloxane; SAM: self-assembled monolayer; ST: switch-tag; TCEP: Tris(2-carboxyethyl)phosphine hydrochloride; TEG: triethyleneglycolundecane; TGF: transforming growth factor; VEGF: vascular endothelial growth factor.

## Authors' contributions

JHL, AWM and MAB conceived the study and designed the experiments. BTC designed and engineered the constructs. EAM performed all the experiments and drafted the manuscript. All authors edited, read and approved the final manuscript.
